# Supplementary material for: Monitoring the binding and insertion of a single transmembrane protein by an insertase
Source: Nat Commun. 2021 Dec 6;12:7082. doi: 10.1038/s41467-021-27315-3 (PMC8648943; doi:10.1038/s41467-021-27315-3)
Supplement: Supplementary file 1 — Supplementary Information [file 41467_2021_27315_MOESM1_ESM.pdf]

Supplementary Information

Monitoring the binding and insertion of a single transmembrane protein by an insertase

Pawel R. Laskowski[1], Kristyna Pluhackova[1], Maximilian Haase[2], Brian M. Lang[1], Gisela Nagler[2], Andreas Kuhn[2] & Daniel J. Müller[1,*]

[1]Department of Biosystems Science and Engineering, ETH Zurich, 4058 Basel, Switzerland;

[2]Molecular Microbiology, Biology Institute, Universität Hohenheim, 70599 Stuttgart, Germany.

***Correspondence**: daniel.mueller@bsse.ethz.ch

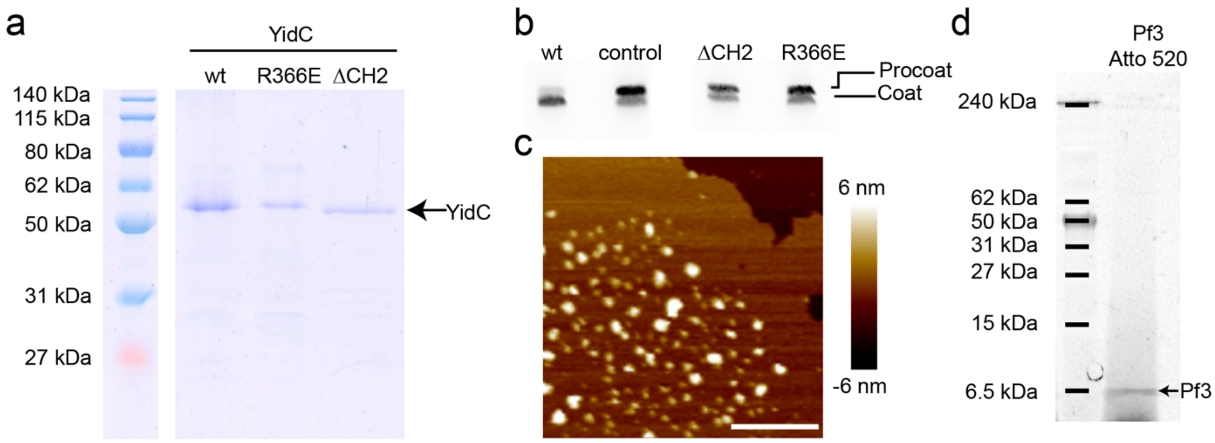

Supplementary Figure 1. Purification and functional analysis of wt, ΔCH2 and R366E YidC. (a) wt YidC, mutant R366E YidC, and mutant ΔCH2 YidC purified from *E. coli* and reconstituted in POPE:POPG lipid membranes. **(b)** *In vivo* insertion assay of YidC. Only after insertion by YidC into the inner membrane of *E. coli* the M13 procoat protein is cleaved to the mature coat[1]. The intensity of the cleaved band (coat) indicates the YidC insertion efficiency. **(c)** Atomic force microscopy (AFM) topography of reconstituted YidC adsorbed to atomically flat mica show sparsely distributed protein assemblies (bright spots) protruding from the phospholipid membrane. YidC protrudes from the membrane surface by 1.64 ± 0.85 nm (mean ± sd; n = 61, where n are individually measured protrusions). Scale bar, 200 nm. **(d)** Fluorescent gel of the purified Pf3 protein. Each experiment in this figure was repeated at least three independent times.

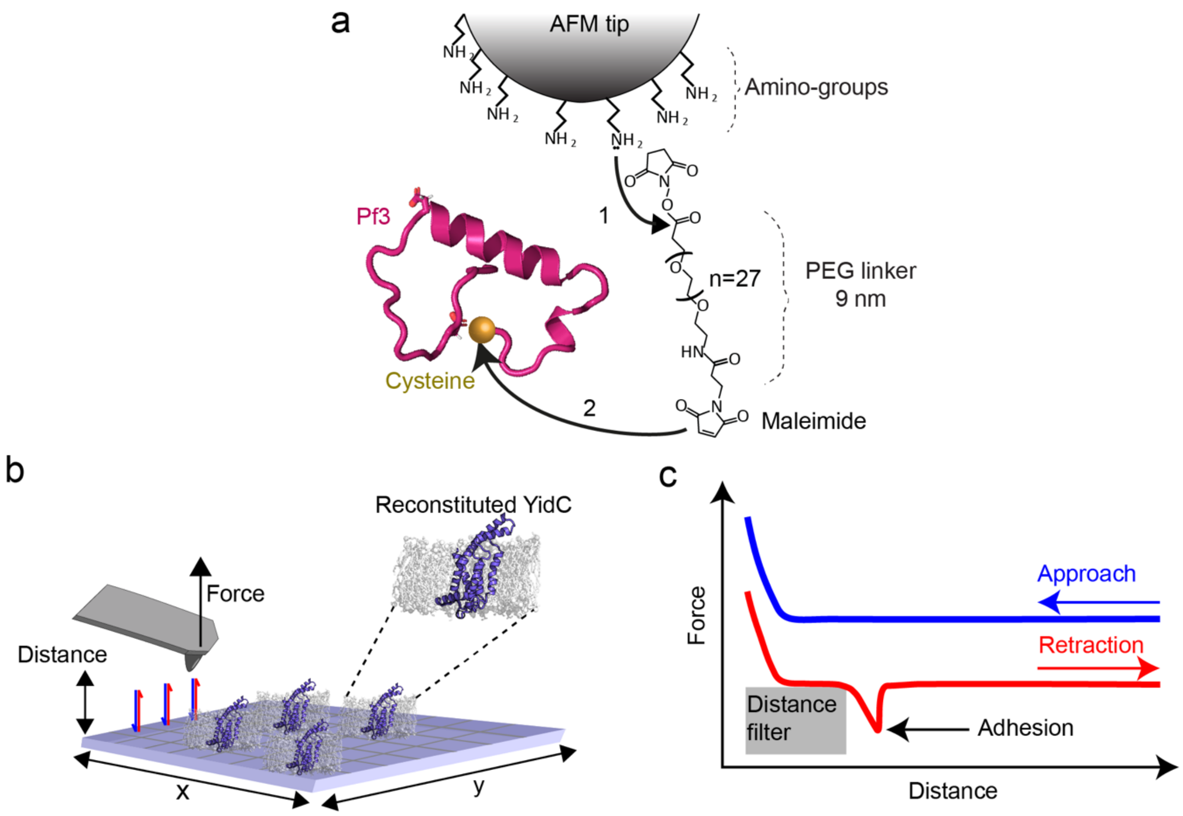

Supplementary Figure 2. Principles of functionalizing the AFM tip by a Pf3 polypeptide and using force-distance curve-based AFM (FD-AFM) to detect specific (un-)binding events between Pf3 covalently tethered to the AFM tip and YidC embedded in a phospholipid membrane. (a) The cysteine-modified C-terminal end of Pf3 is covalently attached to the AFM tip through a heterobifunctionalized polyethylene glycol (PEG_{27}) linker by a sequence of two specific chemical reactions: (1) N-Hydroxysuccinimide (NHS) reacting with amine groups on the AFM tip and (2) maleimide binding to the thiol group of the cysteine at the C-terminus of Pf3. **(b)** For each topographic pixel the Pf3-functionalized AFM tip approaches and retracts from a phospholipid membrane embedding YidC. The sample illustrated here represents YidC in a phospholipid membrane and adsorbed to atomically flat mica (Methods). During each approach and retraction cycle of the Pf3-functionalized AFM tip to and from the sample an approach and retraction force-distance (FD) curve is recorded, respectively (see **c**). The delay time between executing and recording an approach and retraction FD curve is used to adjust the contact time between YidC and Pf3. **(c)** Illustrated approach (blue) and retraction (red) FD curve. The retraction FD curve recorded an adhesive interaction event at a certain distance, corresponding to the stretching of the PEG_{27}-linker tethering the Pf3 to the AFM tip. Thus, applying an appropriate distance filter removes non-specific adhesion events, which usually occur at distances much below the length of the PEG_{27}-linker[2].

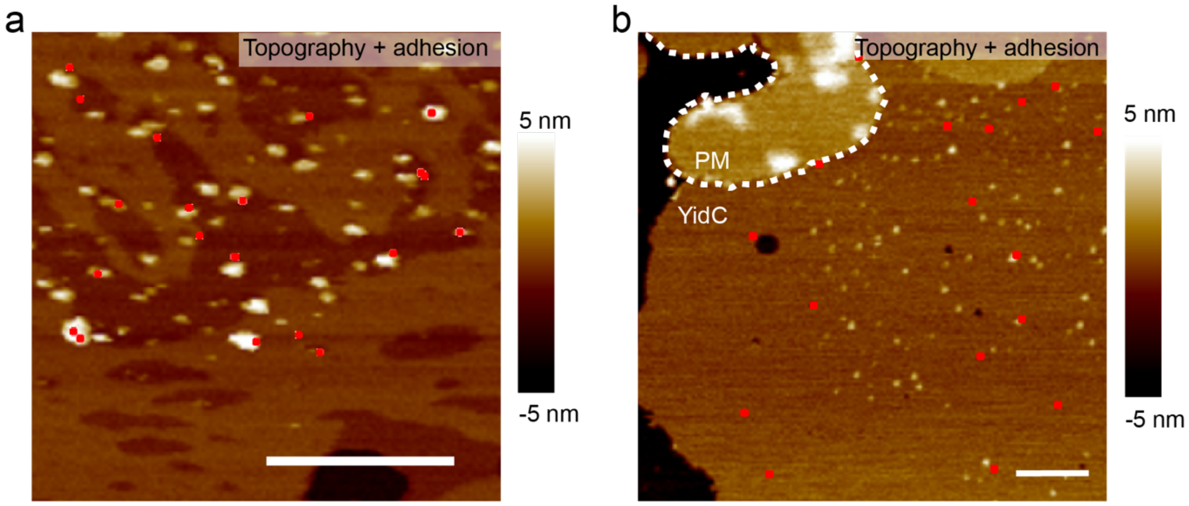

Supplementary Figure 3. Pf3 specifically binds to YidC. (a) FD-AFM topography of YidC in a membrane overlaid with single adhesive interaction events detected in retraction FD curves (red dots). Experiments were performed with a Pf3-functionalied AFM tip as described in Fig. 2 and Supplementary Fig. 2. **(b)** Co-adsorption of a YidC containing membrane and purple membrane (PM) from *Halobacterium salinarum* show the specificity of Pf3 binding to YidC. Purple membrane is densely packed with the light-driven proton pump bacteriorhodopsin and lipids, which makes it suitable to control whether Pf3 undergoes un-specific interactions with another membrane and membrane protein. Each experiment was repeated three independent times. Scale bars, 200 nm.

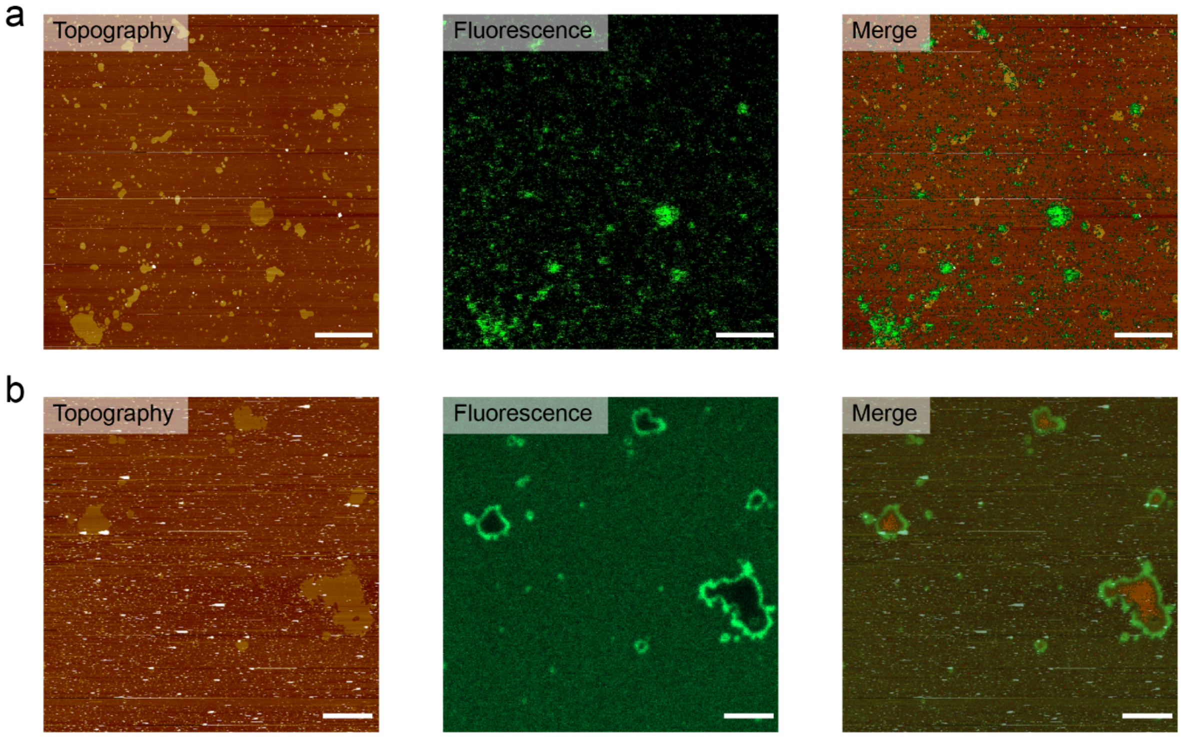

Supplementary Figure 4. Pf3 inserts *via* YidC into supported phospholipid membranes. (a) AFM topography (left), fluorescence image (middle) and superimposed topography and fluorescence image (right) of fluorescently (Atto488) labelled Pf3 and supported phospholipid (POPE:POPG) membrane patches embedding YidC. The fluorescent signal of Atto488-labelled Pf3 co-localizes with the YidC membranes, which indicates Pf3 to be inserted into the membranes. **(b)** AFM topography (left), fluorescence image (middle), and superimposed topography and fluorescence image (right) of fluorescently (Atto488) labelled Pf3 and supported phospholipid (POPE:POPG) membrane patches lacking YidC. AFM topography and fluorescent image show Atto488-labelled Pf3 to surround the YidC-free lipid membranes. Briefly, phospholipid membranes with (**a**) or without (**b**) reconstituted YidC were adsorbed to mica, which had been glued to a microscopy slide, and incubated with Pf3-Atto488 for 30 min in buffer solution (150 mM KCl, 2.5 % isopropanol (v:v), 20 mM Hepes, pH 7.4) at room temperature. The sample topography and fluorescence were imaged using a combined AFM-confocal setup (Methods). Each experimental condition was repeated two independent times. Scale bars, 5 µm.

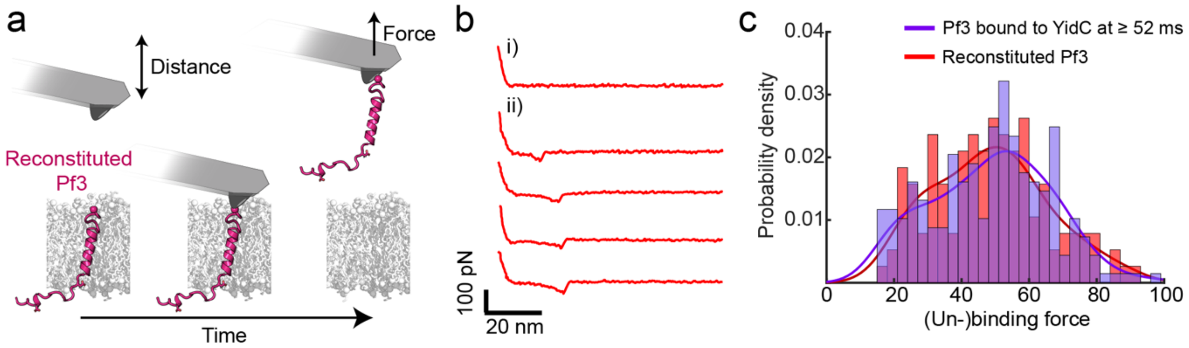

Supplementary Figure 5. Pf3 reaches the membrane inserted state upon 52 ms and 502 ms contact time with YidC reconstituted in phospholipid membranes. **(a)** AFM-based SMFS setup applied to mechanically extract and unfold single Pf3 reconstituted into POPE:POPG lipid membranes (Methods). The bare AFM tip was pushed onto the Pf3 membrane until reaching a force of 150 – 200 pN. The tip was kept in contact with the membrane for 75 – 100 ms and afterwards retracted. In ≈0.34% of all cases (n = 127/37,896) the polypeptide end of a single Pf3 attached non-specifically to the AFM tip and the retracting cantilever extracted the protein from the membrane. **(b)** Example FD curves recorded upon retracting the AFM tip from the membrane. i) Most FD curves (99.66%) show no adhesive interactions of the AFM tip with the membrane. ii) Occasionally, the FD curves show an adhesive force peak at a distance corresponding to the contour length of the fully unfolded and stretched Pf3 polypeptide (≈15 nm) indicating the extraction of the Pf3 polypeptide. **(c)** Histograms of (un-)binding forces of Pf3 from YidC, which were collected using SMFS at extended contact times of 52 ms and 502 ms (purple bars, data taken from Fig. 2c), match the forces characterizing the extraction and unfolding of reconstituted Pf3 (red bars). A two-sided Mann–Whitney U tests estimated no significant difference between both data sets (P = 0.7961). Purple and red curves describe kernel density estimations of the corresponding data sets. Source data are provided as a Source Data file.

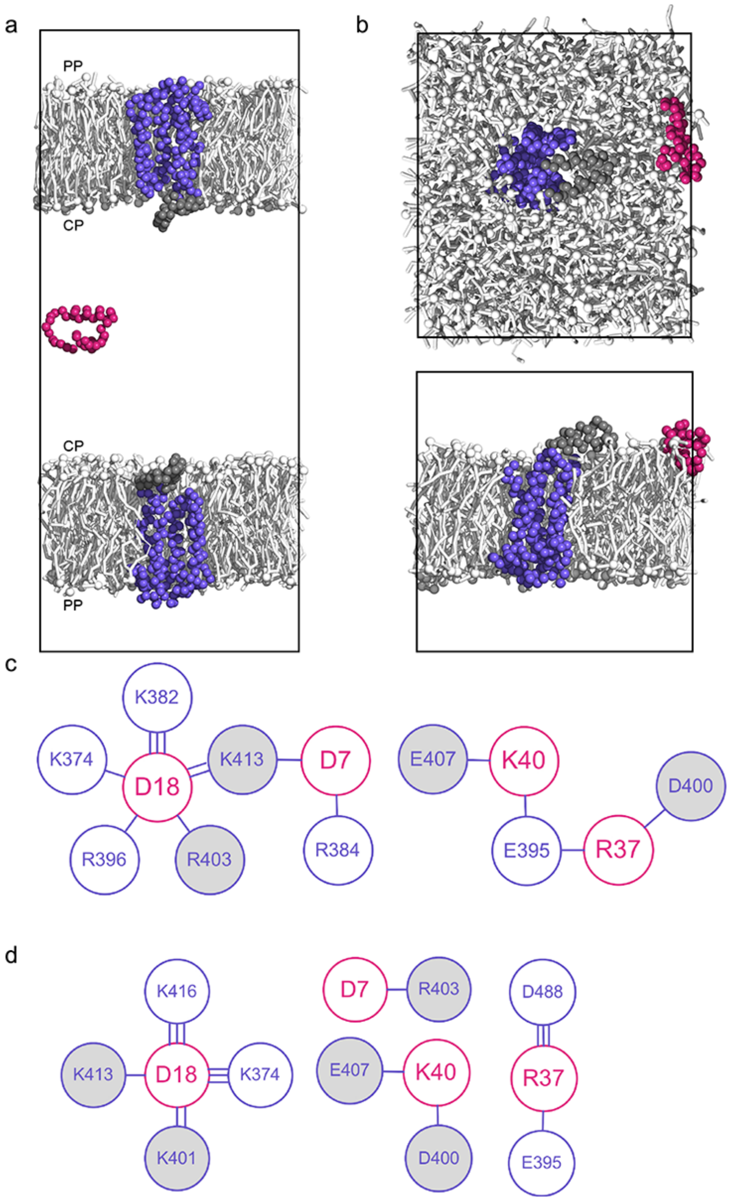

Supplementary Figure 6. Systems used to simulate the spontaneous binding of YidC and Pf3 at coarse-grained (CG) resolution and overview of salt bridges formed in spontaneous binding simulations. (a) Side view of the simulation system containing two lipid membranes having YidC (purple) inserted in mirrored orientations and a Pf3 polypeptide (red) in between. CP denotes cytoplasm, PP periplasm. **(b)** Top-view (upper image) and side-view (bottom image) representation of the simulation system showing Pf3 interacting with the membrane surface. The backbones of all proteins are shown as spheres and colored red for Pf3 and purple for YidC. The two cytoplasmic α-helices of YidC are colored grey. The membrane is shown as white sticks and spheres (phosphate groups). Simulation boxes are framed in black. **(c)** Salt bridges formed between Pf3 (red) and YidC (purple) residues in 10 independent spontaneous binding simulations indicated in (**a**). In each of the simulations Pf3 was initially localized in water. **(d)** Salt bridges established between Pf3 and YidC in 12 independent spontaneous binding simulations indicated in (**b**). In each simulation Pf3 initially interacted with the membrane. Grey shaded circles highlight residues of the cytoplasmic α-helix CH2 of YidC. The number of lines connecting the circles represents how often a particular interaction was formed in the final frame of each simulation.

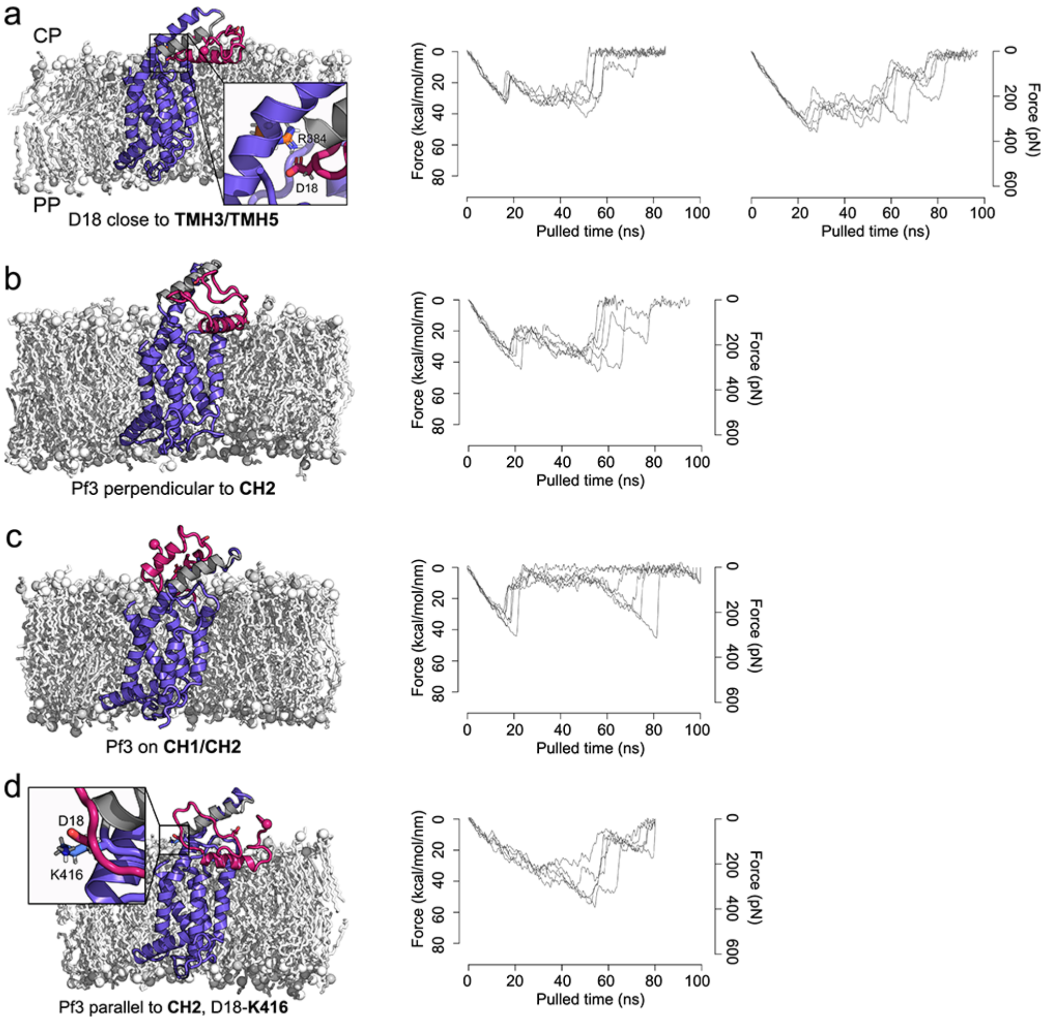

Supplementary Figure 7. Snapshots from molecular dynamics (MD) simulations of Pf3 bound to cytoplasmic α-helices CH1 and CH2 of YidC and force-time (FT) curves describing the mechanical separation of the YidC-Pf3 complexes. (a) Complexed with YidC, Pf3 locates between the cytoplasmic α-helices (CHs) of YidC and the membrane surface. Thereby residue D18 of Pf3 points in the direction of the hydrophilic groove between the transmembrane α-helices (TMH) TMH3 and TMH5 of YidC ("D18 close **to TMH3/TMH5**"). In some YidC-Pf3 complexes (FT curves on the most right) D18 (Pf3) forms a salt bridge with R384 (YidC), shown in an inset, while others (FT curves in the middle) show no salt bridge. CP denotes cytoplasm, PP periplasm. **(b)** In the YidC-Pf3 complex "Pf3 perpendicular to **CH2**", Pf3 localizes between the CH1 and CH2 and the membrane surface. The α-helical part of Pf3 is perpendicular to CH2 and does not form direct contacts with the CHs. Instead, Pf3 contacts YidC via its disordered regions (residues 1–17 and 34–44 of Pf3). **(c)** In the YidC-Pf3 complex "Pf3 on **CH1/CH2**", the α-helical part of Pf3 binds and orients parallel to both cytoplasmic α-helices. The N-terminus of the Pf3 interacts with the loop in between TMH5 and TMH6 of YidC. **(d)** In the YidC-Pf3 complex "Pf3 parallel to **CH2**, D18-**K416**", Pf3 binds between CH2 and the membrane surface with the central α-helical part of Pf3 being parallel to the CH2. D18 (Pf3) stabilizes by forming a salt bridge to K416 (YidC), shown in the inset. Maximal (un-)binding forces of FT curves, which were recorded while mechanically separating the complexes, are plotted in Fig. 3d. YidC residues or α-helices in the complex names are given in bold. Simulation lengths and number of repetitions of the MD simulations are given in Supplementary Table 2. Source data are provided as a Source Data file.

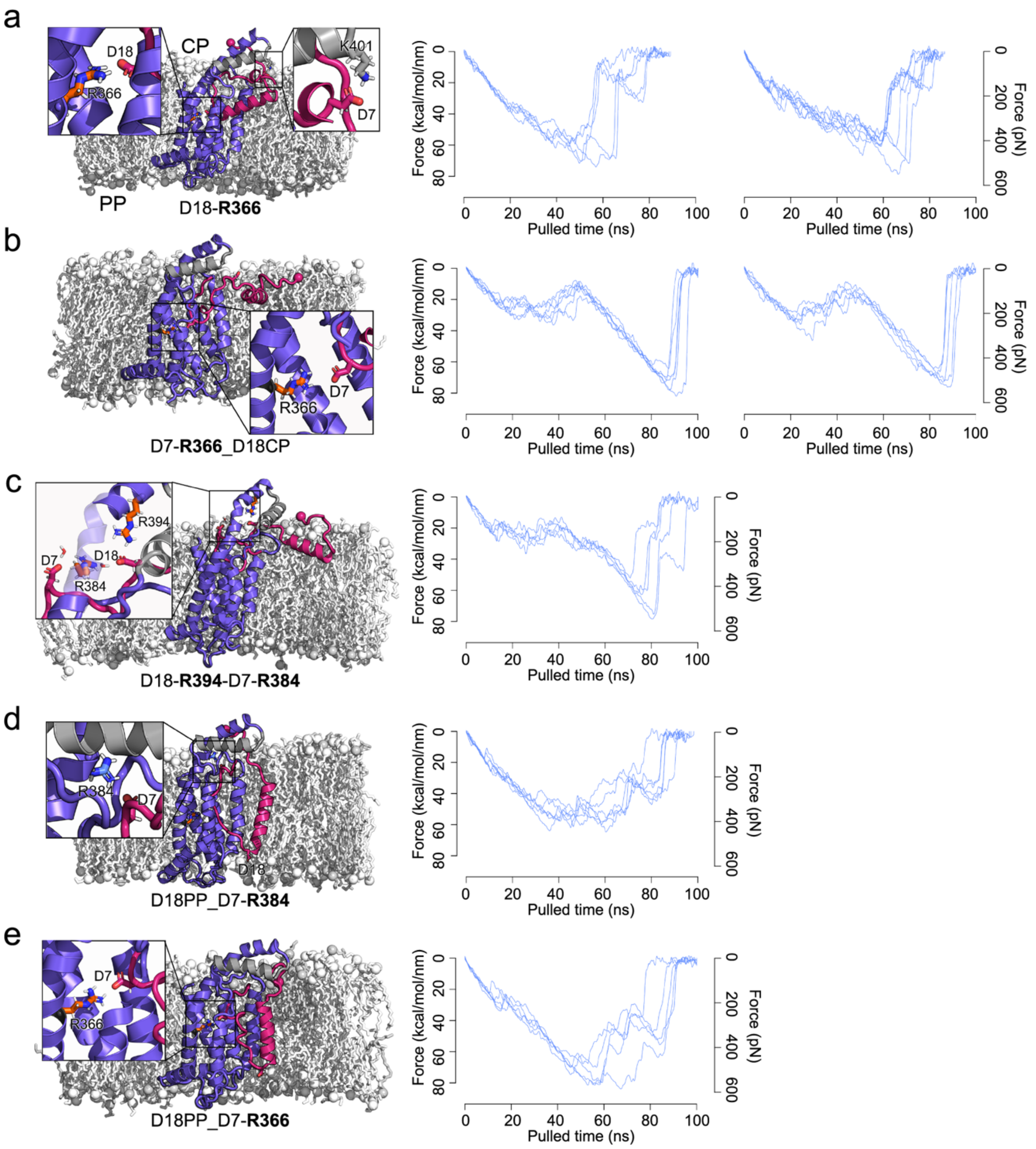

Supplementary Figure 8. Snapshots from MD simulations of Pf3 bound to the hydrophilic groove of YidC and FT curves describing the mechanical separation of the YidC-Pf3 complexes. (a) In the YidC-Pf3 complex "D18-**R366**", Pf3 inserts in the hydrophilic groove of YidC and the α-helical part of Pf3 locates in the bilayer and points between TMH3 and TMH5 of YidC. The deeply buried D18 of Pf3 stabilizes by a salt bridge with R366 of YidC (left inset) and D7 of Pf3 binds to K401 of YidC (right inset). The two graphs show FT curves recorded during separating two different YidC-Pf3 complexes, each of which being obtained by an independent atomistic equilibration of the "D18-**R366**" complex. CP indicates the cytoplasmic side and PP the periplasmic side of YidC. **(b)** In the YidC-Pf3 complex "D7-**R366**_D18CP", the N-terminal loop of Pf3 inserts into the hydrophilic groove and stabilizes by a salt bridge between D7 (Pf3) and R366 of YidC, shown in the inset. The α-helical part of Pf3 inserts and orients parallel to the hydrophilic/hydrophobic membrane interface. D18 of Pf3 interacts with the lipid head groups at the cytoplasmic side. The two plots show FT curves recorded during separating the "D7-**R366**_D18CP" complex after 500 ns (left) and 1,000 ns (right) equilibration simulation at all-atom

resolution. **(c)** In the YidC-Pf3 complex "D18-**R394**-D7-**R384**", the N-terminal segment of Pf3 between D7 and D18 inserts in the hydrophilic groove of YidC. D7 of Pf3 stabilizes by forming interactions with R384 of YidC and D18 of Pf3 is locked in between R384 and R394 of YidC, shown in the inset. The α-helical part of Pf3 inserts and orients parallel to the hydrophilic/hydrophobic membrane interface. **(d)** In the YidC-Pf3 complex named "D18PP_D7-**R384**", the α-helical part of Pf3 adopts a transmembrane orientation. D18 of Pf3 interacts with the lipid head groups at the periplasmic side. The N-terminus of Pf3 locates at cytosolic side and D7 of Pf3 binds to R384 of YidC, highlighted in the inset. **(e)** The YidC-Pf3 complex, entitled "D18PP_D7-**R366**", shows a transmembrane orientation of the α-helical part of Pf3. D18 of Pf3 interacts with the lipid head groups at the periplasmic side. The N-terminus of Pf3 locates at the cytoplasmic entry of the hydrophilic groove of YidC. D7 of Pf3 inserts deeply in the hydrophilic groove and forms a salt bridge with R366 of YidC, shown in the inset. Maximal (un-)binding forces of FT curves which were recorded while mechanically separating the complexes, are plotted in Fig. 3d. YidC residues or α-helices in the complex names are highlighted in bold. Simulation lengths and number of repetitions of the MD simulations are given in Supplementary Table 2. Source data are provided as a Source Data file.

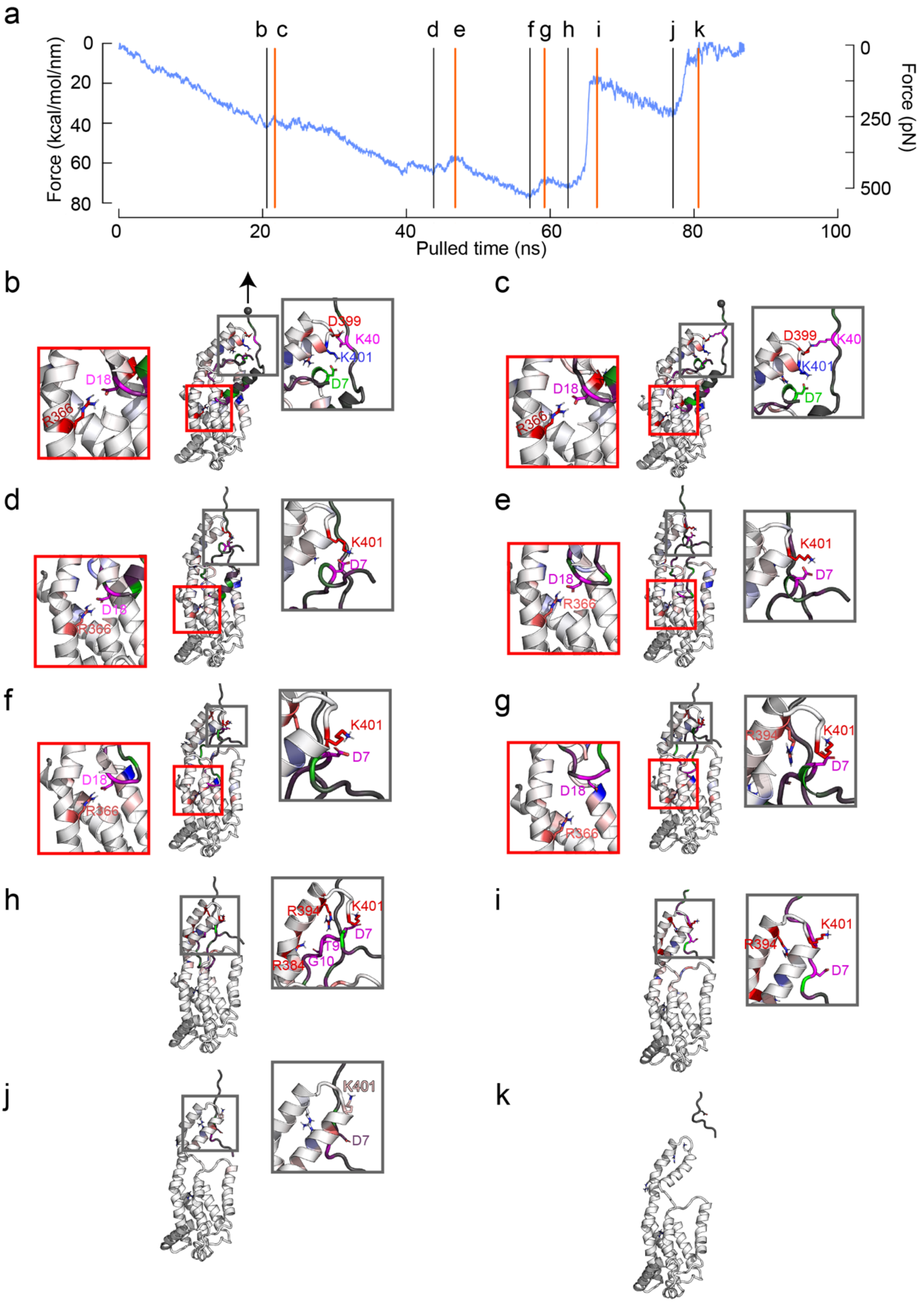

Supplementary Figure 9. Snapshots of a typical force-time curve recorded upon mechanically separating Pf3 from YidC in the D18-R366 complex (Supplementary Fig. 6c) using MD simulations. **(a)** Force-time curve with the local force minima and maxima labelled and their structural snapshots shown in **(b-k).** **(b)** Upon pulling on the C terminus of Pf3 (highlighted by a sphere) the peptide is slowly stretched, while all three salt bridges stabilizing the complex, D18-**R366**, D7-**K401** and K40-**D399,** remain intact. The arrow indicates the pulling direction. **(c)** Further pulling stresses the K40-**D399** salt bridge which ruptures immediately afterwards. **(d)** While D7-**K401** and D18-**R366** salt bridges remain

intact, the α-helical part of Pf3 begins to unfold. **(e)**. While the two salt bridges, D7-**K401** and D18-**R366**, are still stable, the α-helix is completely unfolded. **(f)** At the global maximum of the (un-)binding force, the force acts directly on D18-**R366.** **(g)** Rupture of the D18-**R366** salt bridge and stepwise removal of Pf3 from the hydrophilic binding groove. **(h)** Pf3 rebinds YidC *via* a quickly binding intermediate comprising a salt bridge between D7 and **K401** and a number of hydrogen bonds between the cytoplasmic α-helices (**R384, R394**) and the N-terminal part of Pf3 (G10, T9). **(i)** Finally, the salt bridge between D7 and **K401** ruptures. **(j)** Only weak forces act between the N-terminal end of Pf3 and the cytoplasmic α-helices of YidC, D7 and **K401** do not form a salt bridge anymore. **(k)** Pf3 is separated from YidC. YidC residues are highlighted in bold. For visualization of the full pulling process, see Supplementary Movie 1.

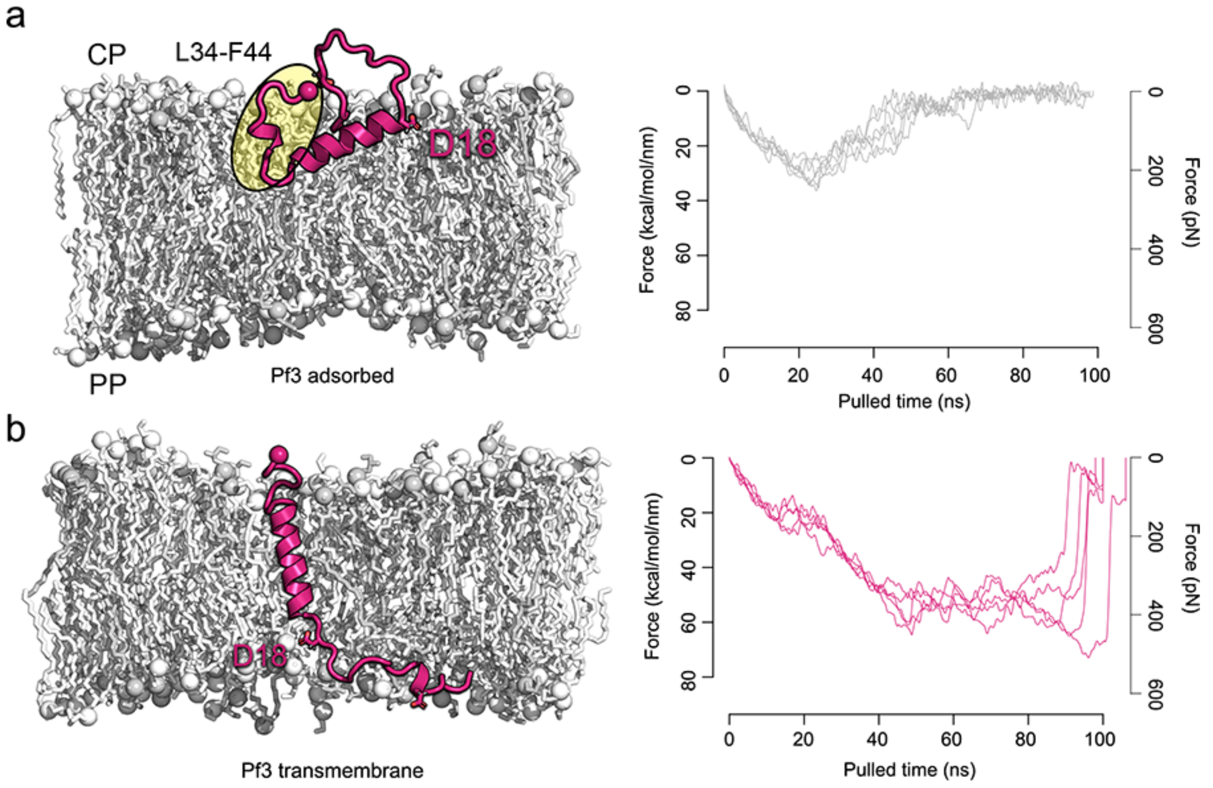

Supplementary Figure 10. Snapshots of MD simulations of Pf3 adsorbed to and inserted into the phospholipid membrane and FT curves describing the mechanical separation of Pf3 from the membrane. (a) Pf3 adsorbed onto the membrane. The membrane adsorbed state is characterized by the partial insertion of the Pf3 α-helix into the hydrophilic/hydrophobic interface of the membrane. Parts of the C terminal part of the Pf3 polypeptide (residues L34–F44, yellow shaded) are membrane inserted while the negatively charged D18 drives the N-terminal region closer to the membrane/water interface. The FT curves, shown on the right, were recorded during mechanical separation of the Pf3 polypeptide from the membrane interface. **(b)** Pf3 inserted in the transmembrane orientation. The transmembrane α-helix is slightly titled relative to the membrane plane. The FT curves, shown on the right, were recorded during extraction of the Pf3 polypeptide from the membrane. The maximal extraction forces recorded in all FT curves are plotted in Fig. 3d. Simulation lengths and number of repetitions of the MD simulations are given in Supplementary Table 2. CP denotes cytoplasm, PP periplasm. Source data are provided as a Source Data file.

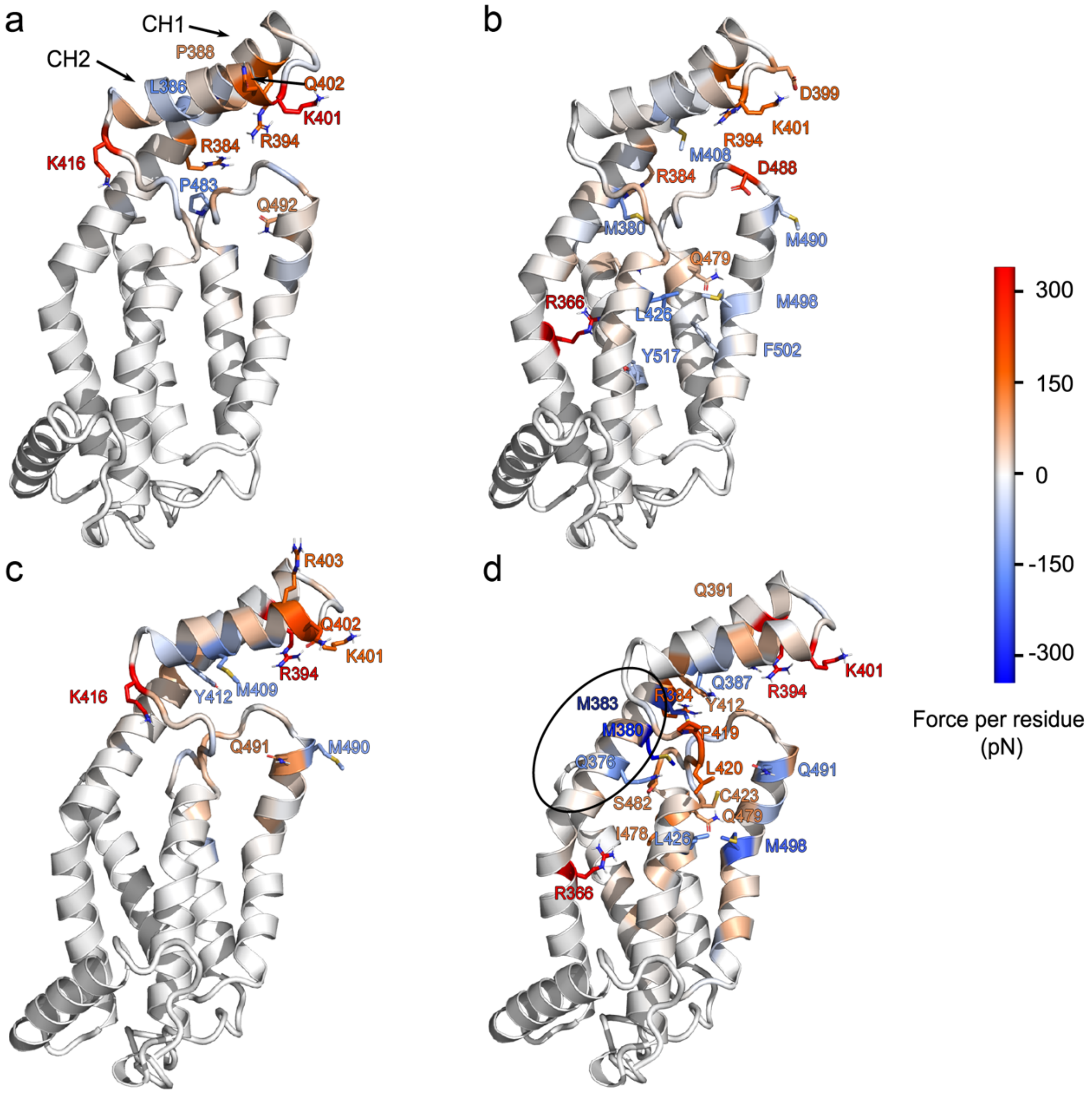

Supplementary Figure 11. Force distribution analysis (FDA) of MD simulations suggests YidC residues that are important for Pf3 binding. (a) Force distribution exerted by Pf3 in the "CH1/CH2 bound" complexes with YidC as found in equilibration simulations. Positively charged YidC residues K401, K416, R384, and R394 together with P388 and Q492 establish attractive (red) interactions with the Pf3 polypeptide. Residues repelling (blue) the Pf3 polypeptide are L386 and P483. Cytoplasmic α-helices CH1 and CH2 are pointed with arrows. **(b)** Force distribution exerted by Pf3 bound to the hydrophilic groove of YidC as found in equilibration simulations. A number of positively charged YidC residues R366, R384, R394, and K401, two negatively charged residues D399 and D488, and the neutral residue Q479 exert attractive forces on the Pf3 polypeptide. The attractive forces are counterbalanced by repulsive forces from methionines, Y517, L426, and F502, assuring that Pf3 stays in place. **(c)** Upon mechanically separating Pf3 from the cytoplasmic α-helices CH1 and CH2 diverse positively charged residues (K401, R394, R403, and K416) as well as Q402 and Q491 attract Pf3, while M409, M490, and Y412 repel Pf3. **(d)** Upon mechanically separating of Pf3 from the hydrophilic groove of YidC, the following three insertase regions exert strong forces on Pf3: (1) residues in the hydrophilic groove (especially R366), (2) residues between TMH2 and CH1 (highlighted by a black ellipse), and (3) residues in the CH1 and CH2. YidC residues establishing attractive forces with Pf3 are colored red and repulsive forces blue. YidC residues not exerting significant forces with Pf3 are colored white. Pf3 was omitted from the visualization to more clearly show the YidC residues involved in Pf3 binding.

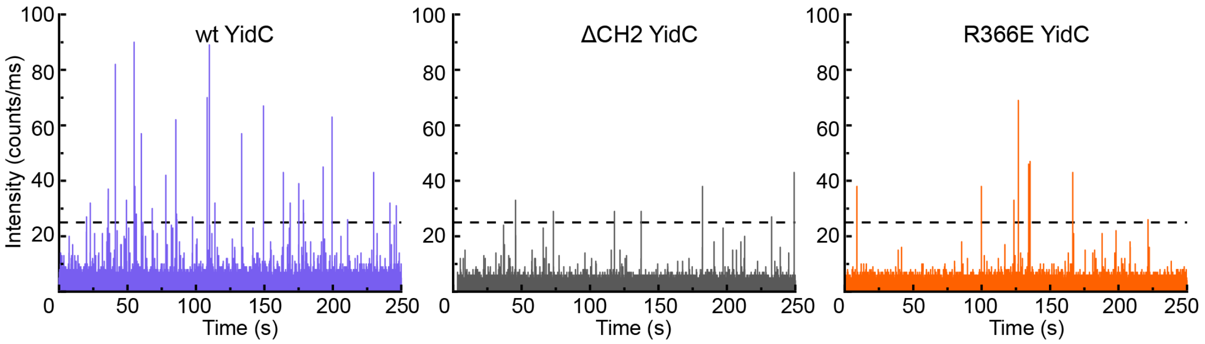

Supplementary Figure 12. Fluorescent time traces of Pf3 insertion into YidC proteoliposomes. The Atto520 dye attached to the N-terminal end of Pf3 is quenched outside the proteoliposomes. However, upon inserting Pf3 into the YidC proteoliposomes the dye shows fluorescence activity, which is measured as fluorescence intensity peaks. The fluorescence intensities recorded by a photodiode were quantified per ms. The resulting fluorescent time traces are used to compare the Pf3 insertion efficiency of wt YidC (purple), ΔCH2 YidC (grey) and R366E YidC (orange), as presented in Fig. 4a,b. The minimum Atto520 intensity of 25 counts/ms (marked as a cutoff with a dashed line) and a diffusion time of at least 40 ms were used for the evaluation of each insertion burst. Compared to wt YidC, both YidC mutants show significantly fewer insertion events. Source data are provided as a Source Data file.

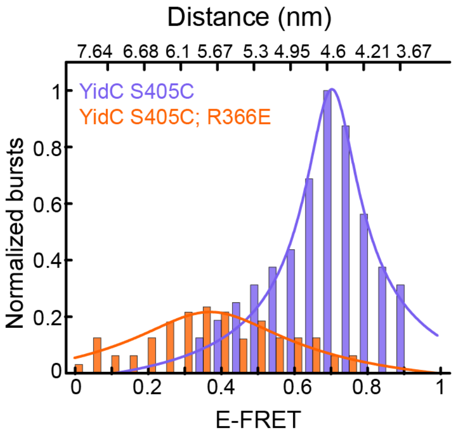

Supplementary Figure 13. Förster resonance energy transfer (FRET) histograms of Pf3 binding to wild-type (wt) YidC S405C and mutant R366E YidC S405C. The Pf3 coat protein was labelled with the donor dye (Atto520) on the C-terminal end (47C), while YidC was labeled with the acceptor dye (Atto647N) in the CH2 region (S405C). Pf3 was mixed with solution of YidC proteoliposomes in equimolar concentration and the measurement, which started immediately after mixing the proteins was continued for 360 s. FRET efficiencies calculated for each individual burst are shown for wt YidC S405C (purple) and mutant R366E YidC S405C (orange). About four times less FRET events occurred for R366E YidC compared to wt YidC in the same measurement period (360 s). wt YidC and R366E YidC show an average distance to the Atto520 dye of Pf3 of 4.6 nm and 5.8 nm, respectively. Histograms are fitted with a Gaussian distribution. Förster distances were calculated based on the calculated average FRET efficiency (E-FRET) values for single bursts of fluorescence. Source data are provided as a Source Data file.

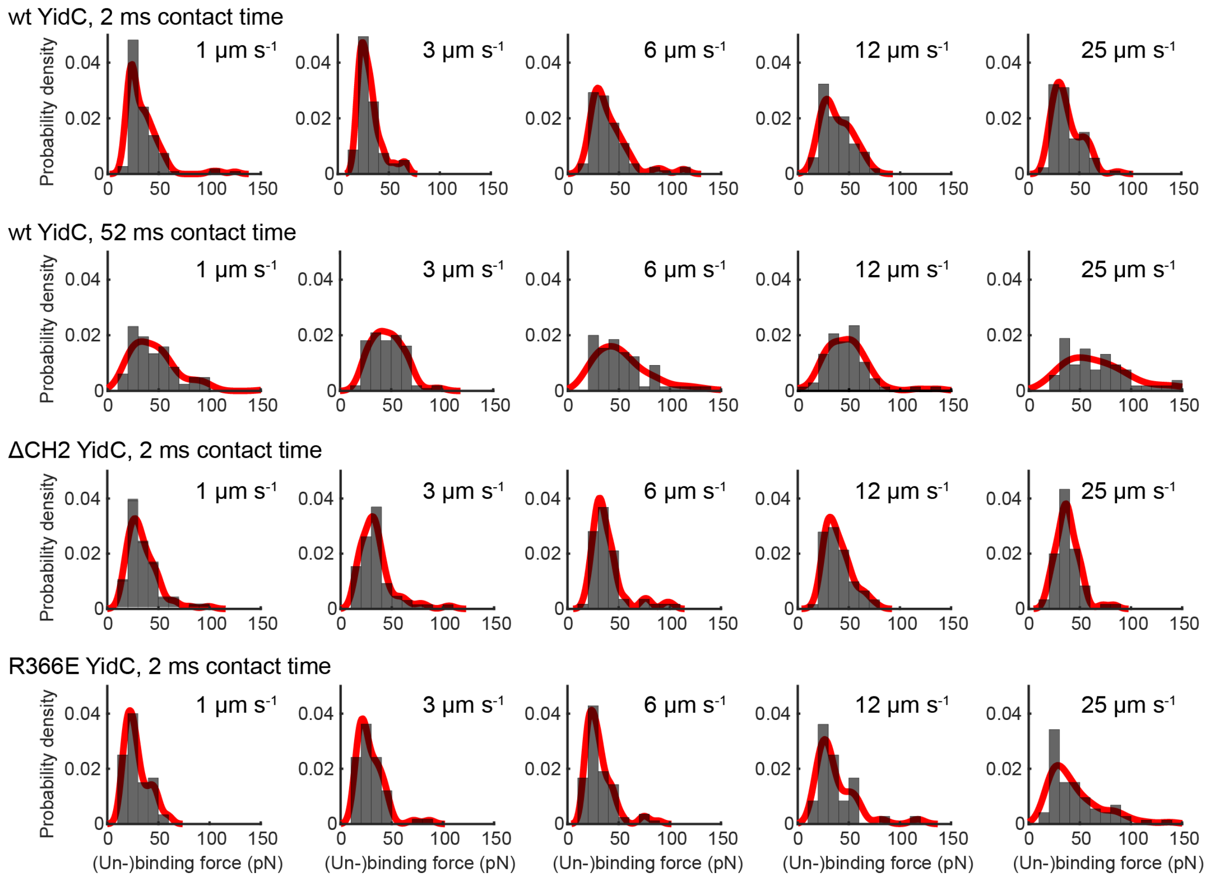

Supplementary Figure 14. Histograms of (un-)binding forces collected during dynamic force spectroscopy (DFS) experiments together with their kernel density estimations. (Un-)binding forces of Pf3 from wt YidC, mutant ΔCH2 YidC, and mutant R366E YidC, which were collected at different pulling velocities (grey bars) were fitted with kernel density estimation (red lines) to calculate the most probable (un-)binding force and the most probable loading rate for each pulling velocity. Contact times are indicated. Extracted values were fitted using the Bell-Evans model (Fig. 5). Source data are provided as a Source Data file.

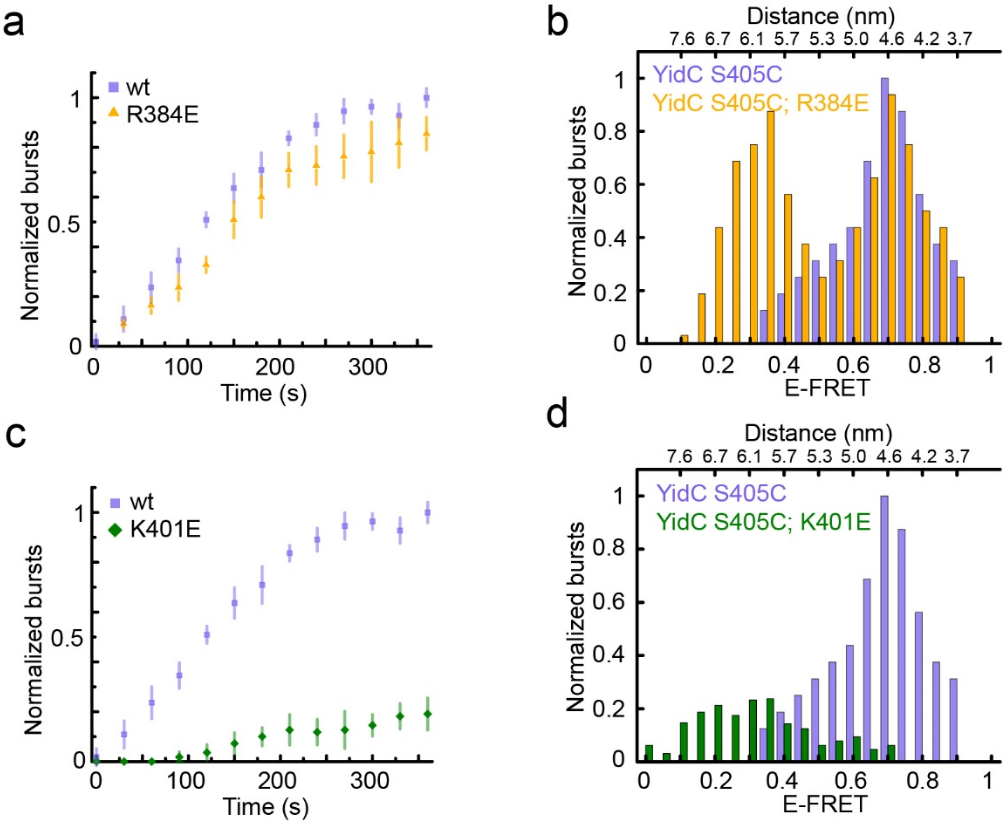

Supplementary Figure 15. Single mutations in the cytoplasmic helices CH1 and CH2 of YidC affecting the binding and insertion of Pf3. (a) Pf3 insertion into mutant R384E (yellow) and wild-type (wt) (purple) YidC proteoliposomes as measured by FCS. The Atto520 dye attached to the N-terminal end of Pf3-3C (Methods) is quenched outside proteoliposomes and bursts fluorescence upon translocation *via* YidC into proteoliposomes. Data points represent means from 35 measurements and error bars sd. **(b)** FRET histograms of Pf3 binding to wt YidC S405C and mutant R384E YidC S405C. FRET efficiencies calculated for each individual burst are shown for wt YidC S405C (purple) and mutant R384E YidC S405C (yellow). Förster distances were calculated based on the calculated average FRET efficiency (E-FRET) values for single bursts of fluorescence. **(c)** Pf3 insertion into wt YidC S405C (purple) and mutant K401E YidC S405C (green) proteoliposomes as measured by FCS. Data points represent means from 35 measurements and error bars sd. **(d)** FRET histograms of Pf3 binding to wt YidC S405C and mutant K401E YidC S405C. Source data are provided as a Source Data file.

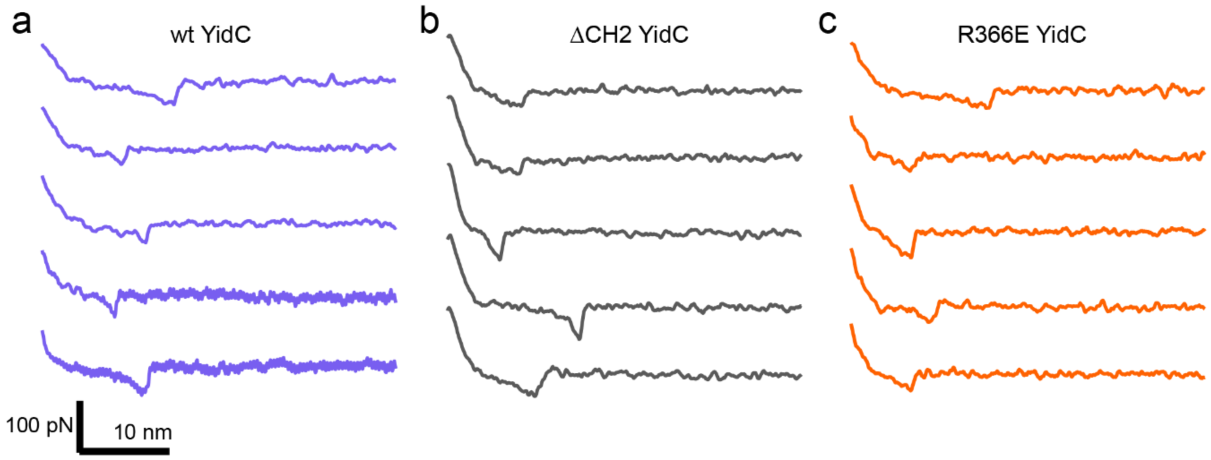

Supplementary Figure 16. FD curves detected upon (un-)binding of Pf3 from YidC. The exemplified retraction FD curves show the unbinding of Pf3 from **(a)** wt YidC (purple), **(b)** ΔCH2 YidC (grey) and **(c)** R366E YidC (orange). Individual FD curves reveal similar single adhesive unbinding events of Pf3 from wt YidC and the YidC mutants. The C-terminal end of Pf3 had been covalently tethered to the tip of the AFM cantilever as described (Supplementary Fig. 2). FD curves were taken from the experimental data presented in Fig. 2c, 4c,d collected at 2 ms contact time. Source data are provided as a Source Data file.

Supplementary Methods

Generation of YidC-Pf3 complexes

At first, Pf3 was let to spontaneously bind to YidC in coarse-grained simulations. Therefore, Pf3 was localized in the aqueous solution and a double simulation system was prepared in which the cytoplasmic surfaces of both YidC faced the same water slab into which Pf3 has been placed (Supplementary Fig. 6a). Thus, Pf3 binding to the periplasmic side of YidC was avoided. In all ten spontaneous binding simulations, Pf3 bound to the outer cytoplasmic region of YidC far from the entry to the hydrophilic groove. The α-helix of Pf3 oriented twice perpendicularly to CH1 and CH2, four times parallel to CH1 and CH2, and four times antiparallel to CH1 and CH2. The position of Pf3 was stabilized by 1–3 salt bridges (Supplementary Fig. 6c). One of the complexes formed was exemplarily converted to atomistic resolution to test for its stability and to quantify the force required to separate the YidC-Pf3 complex. In the next step, Pf3 was placed close to the membrane surface enabling it to adsorb to the membrane before contacting YidC (Supplementary Fig. 6b). Within 5–10 μs of CG simulations in 7 out of 12 simulations the α-helical part of Pf3 localized between CH1, CH2 and the membrane surface in diverse orientations stabilized by diverse salt bridges with YidC (Supplementary Fig. 6d). In four simulations D18 of Pf3 attached to K416 (three times via salt bridges) of YidC and once R37 of Pf3 formed a salt bridge with D488 of YidC, with the α-helical part of Pf3 pointing away from YidC. One of the complexes with the lowest interaction energy between Pf3 and YidC was converted to atomistic resolution and re-equilibrated atomistically.

In order to increase the probability of Pf3 to enter the hydrophilic groove of YidC we have performed a replica exchange tempering simulation (using 9 replicas simulated at temperatures ranging from 298 K till 538 K with 30 K steps, 1 μs CG simulation each), tempering both proteins (Pf3 and YidC) and the membrane while keeping the membrane area constant. The analysis of the replica at 298 K revealed that in one third of the 100,000 analyzed YidC-Pf3 complexes Pf3 did not interact with YidC, whereas in 60% of the complexes Pf3 interacted weakly with YidC (in 87% of the complexes Pf3 attached to YidC by a single salt bridge between D18 and K493). 6% of the 100,000 analyzed complexes established strong interaction energies (< –350 kJ/mol) between Pf3 and YidC. 99.6% of the complexes stabilized by strong interaction energies (< –350 kJ/mol) formed a salt bridge between D18 of Pf3 and R394 of YidC, and the α-helix of Pf3 (residues D18–G35) localized between both CHs of YidC and the membrane. Thereby, Pf3 adopted two slightly different conformations. In the first configuration, comprising 98.7 % of the YidC-Pf3 complexes, the Pf3 α-helix was closer to CH1 and CH2 of YidC, while in the second configuration comprising 1.3% of the complexes (in total 77 structures), the Pf3 α-helix inserted deeper into the membrane. A representative complex from the most populated cluster of orientations (containing 5,819 out of 6,039 complexes) was converted back to atomistic resolution and equilibrated atomistically. During the atomistic simulation the α-helical part of Pf3 inserted deeper into the lipid membrane as compared to the CG structure leading to the disruption of the salt bridge between D18 (Pf3) and R394 (YidC). Although the simulations delivered YidC-Pf3 complexes showing Pf3 in a conformation convenient to entry the hydrophilic groove of YidC, a spontaneous entry into the hydrophilic groove was not observed. We hence pulled one of the negatively charged residues, D7 or D18, into the hydrophilic groove of YidC in different depths. The conformations were equilibrated at CG resolution for 1 – 50 μs before converting them to atomistic resolution and re-equilibration at atomistic resolution. The simulations resulted in a complex with D7 or D18 of Pf3 bound to R366 or R384 of YidC. In the final step, D18 was pulled from R366 to the phosphates on the periplasmic side of the bilayer and two resulting conformations (one with D7 attached to R366 and one with D7 on the cytoplasmic side of the membrane) were equilibrated atomistically.

For full list of MD simulations performed at coarse-grained resolution see Supplementary Table 3.

Simulation conditions

In all Martini simulation, the recommended simulation input parameters for the polarizable Martini2.2p in Gromacs 5 and higher were used[4]. In detail, the temperature was kept at 298 K by velocity rescale thermostat[5] and if not stated otherwise the pressure was controlled by the Berendsen barostat[6] in semiisotropic manner to be at 1 bar, using 12 ps time constant and compressibility of $3x10^{-4}$ bar^{-1}. The integration step amounted to 20 fs. The Verlet cut-off scheme[7] was used for neighbor search and the electrostatic interactions behind 1.1 nm cutoff were described by particle mesh Ewald (PME)[8]. The van der Waals forces were switched to zero at 1.1 nm over the full interaction length using the Potential-shift-Verlet modifier[7].

The atomistic simulation parameters followed our earlier recommendations[9,10]. In detail, the time step of 2 fs was used, the center of mass motion of the system was linearly removed every 100 steps. The Verlet cut-off scheme[7] was used for neighbor-search with buffer tolerance of 0.005 kJ mol^{-1} ps^{-1} per atom. The electrostatics behind 1.2 nm cutoff was described by PME[8], the 12-6 Lennard-Jones potential was switched to zero between 0.8 and 1.2 nm using the potential-switch function. The temperature was controlled to remain at 298 K by the Nosé-Hoover[11] thermostat using the time constant of 0.5 ps and in the isothermal-isobaric (NpT) ensembles the pressure was controlled in semiisotropic manner by the Parrinello-Rahman barostat[12,13] with the time constant of 5 ps and compressibility of $4.5x10^{-5}$ bar^{-1} to be 1 bar.

Setup of pulling simulations

For pulling simulations a solvent slab had to be added to the equilibrated system so that the box size was large enough to comprise a fully stretched Pf3 (about 27 nm in the pulling direction). These enlarged simulation boxes were re-equilibrated for 10 ns while keeping the membrane area constant and the velocities from the original simulation. Production run pulling simulations were run for 100 ns each, using pulling rate 0.2 m s^{-1}, umbrella force constant of 0.1 N m^{-1}, at 298 K in the canonical (NVT) ensemble. Thereby the backbone of F44 (Pf3) was pulled away from the center of mass of YidC and the membrane along the membrane normal (using the routine direction-periodic). Each repetition (at least 5 per each YidC-Pf3 complex type) were initiated generating different atom velocities. For full list of performed MD simulations at all-atom resolution see Supplementary Table 2.

Contact time (ms)	wt (pN; mean ± sd)	ΔCH2 (pN; mean ± sd)	R366E (pN; mean ± sd)	wt *vs*. ΔCH2 *P*-value	wt *vs*. R366E *P*-value	*ΔCH2 vs. R366E P-value*
2	29.5 ± 12.4	35.4 ± 16.8	29.1 ± 13.3	0.0148	0.6997	0.0181
3	34.7 ± 14.5	32.5 ± 12.6	27.0 ± 11.6	0.2844	0.0004	0.0028
4	33.7 ± 13.6	38.6 ± 19.6	26.3 ± 12.0	0.1824	<0.0001	<0.0001
7	40.1 ± 18.1	34.6 ± 17.5	29.6 ± 14.7	0.0139	<0.0001	0.0154
12	39.6 ± 16.0	35.5 ± 13.0	31.4 ± 18.2	0.0934	0.0001	0.0064
22	41.6 ± 15.4	36.4 ± 13.5	34.1 ± 17.9	0.0042	<0.0001	0.0308
52	47.6 ± 17.3	41.0 ± 15.3	31.5 ± 12.3	0.0009	<0.0001	<0.0001

Supplementary Table 1. Summary of mean (un-)binding forces of the YidC-Pf3 complex as measured by SMFS (Fig. 4c-e) and their statistical analysis. Force distributions were statistically compared (*P*-values) with a Mann–Whitney U test. Source data are provided as a Source Data file.

**Complex name (Pf3-YidC)**	**Complex group**	**Equilibration**	**Pulling repetitions 100 ns each v = 0.2 m s^{-1}**
D18-**R366**	Pf3 bound in the hydrophilic groove of YidC	2 x 1 µs	4 (after 300 ns sim1)<br>4 (after 1,000 ns sim1)<br>5 (after 1,000 ns sim2)
D18PP_D7-**R384**		1 µs	6
D18PP_D7-**R366**		1 µs	5
D7-**R366**_D18CP		1 µs	6 after 1,000 ns equilibration<br>5 after 500 ns equilibration
D7-**R384**-D18-**R394**		1 µs	5
Pf3 transmembrane	Pf3 in transmembrane orientation	300 ns	6
Pf3 adsorbed	Pf3 adsorbed on the membrane surface	200 ns	6
D18 close to **TMH3/TMH5**	Pf3 bound to the cytoplasmic domains of YidC	600 ns, 1 µs	5 after 600 ns sim1<br>5 after 1,000 ns sim2
Pf3 on **CH1/CH2**		200 ns	6
Pf3 parallel to **CH2**, D18-**K416**		500 ns	5
Pf3 perpendicular to **CH2**		500 ns	5
Total simulation time		9.3 µs	7.8 µs

Supplementary Table 2. Summary of performed simulations at all-atom resolution. The complex naming includes specific parts and residues of Pf3 (normal text) and of YidC (bold text) and the names are the same as in Supplementary Fig. 7, 8, 10. CP and PP in complex names stand for cytoplasmic and periplasmic side of the membrane, respectively. CH1 and CH2 denote the cytoplasmic α-helix 1 and 2 of YidC, respectively. TMH3 and TMH5 stand for transmembrane α-helix 3 and 5 of YidC, respectively.

Initial state	Simulation type	Simulation length and number of repetitions
Pf3 in solution, double membrane setup with one YidC per membrane	Pf3 spontaneous binding to YidC from solution	9x1µs, 3µs
Membrane adsorbed Pf3	Pf3 spontaneous binding to YidC from the membrane surface	10x5µs, 2x10µs<br>REST (9x1µs)
D18(Pf3)-**R366(YidC)**	Complex equilibration	2x25µs, 20µs, 50µs
D18(Pf3)-**R384(YidC)**	Complex equilibration	2x25µs, 5µs
D7(Pf3)-**R366(YidC)**	Complex equilibration	5µs
D7(Pf3)-**R384(YidC)**	Complex equilibration	5µs
D18(Pf3)PP_D7(Pf3)-**R384(YidC)**	Complex equilibration	5µs

Supplementary Table 3. Summary of all performed simulations at coarse-grained resolution. REST stands for replica exchange solute tempering, PP for periplasmic side. Actual simulation times are given.

Supplementary References

1 Samuelson, J. C. *et al.* Function of YidC for the insertion of M13 procoat protein in Escherichia coli: translocation of mutants that show differences in their membrane potential dependence and Sec requirement. *J. Biol. Chem.* **276**, 34847-34852, doi:10.1074/jbc.M105793200 (2001).

2 Medalsy, I., Hensen, U. & Muller, D. J. Imaging and Quantifying Chemical and Physical Properties of Native Proteins at Molecular Resolution by Force–Volume AFM. *Angew. Chem. Int. Ed.* **50**, 12103-12108, doi:10.1002/anie.201103991 (2011).

3 Evans, E. & Ritchie, K. Dynamic strength of molecular adhesion bonds. *Biophys. J.* **72**, 1541-1555, doi:https://doi.org/10.1016/S0006-3495(97)78802-7 (1997).

4 de Jong, D. H., Baoukina, S., Ingólfsson, H. I. & Marrink, S. J. Martini straight: Boosting performance using a shorter cutoff and GPUs. *Comput. Phys. Commun.* **199**, 1-7, doi:https://doi.org/10.1016/j.cpc.2015.09.014 (2016).

5 Bussi, G., Donadio, D. & Parrinello, M. Canonical sampling through velocity rescaling. *J. Chem. Phys.* **126**, 014101, doi:10.1063/1.2408420 (2007).

6 Berendsen, H. J. C., Postma, J. P. M., van Gunsteren, W. F., DiNola, A. & Haak, J. R. Molecular dynamics with coupling to an external bath. *J. Chem. Phys.* **81**, 3684-3690, doi:10.1063/1.448118 (1984).

7 Páll, S. & Hess, B. A flexible algorithm for calculating pair interactions on SIMD architectures. *Comput. Phys. Commun.* **184**, 2641-2650, doi:https://doi.org/10.1016/j.cpc.2013.06.003 (2013).

8 Darden, T., York, D. & Pedersen, L. Particle mesh Ewald: An N·log(N) method for Ewald sums in large systems. *J. Chem. Phys.* **98**, 10089-10092, doi:10.1063/1.464397 (1993).

9 Pluhackova, K. *et al.* A Critical Comparison of Biomembrane Force Fields: Structure and Dynamics of Model DMPC, POPC, and POPE Bilayers. *J. Phys. Chem. B* **120**, 3888-3903, doi:10.1021/acs.jpcb.6b01870 (2016).

10 Sandoval-Perez, A., Pluhackova, K. & Böckmann, R. A. Critical Comparison of Biomembrane Force Fields: Protein–Lipid Interactions at the Membrane Interface. *J. Chem. Theory Comput.* **13**, 2310-2321, doi:10.1021/acs.jctc.7b00001 (2017).

11 Evans, D. J. & Holian, B. L. The Nose–Hoover thermostat. *J. Chem. Phys.* **83**, 4069-4074, doi:10.1063/1.449071 (1985).

12 Parrinello, M. & Rahman, A. Crystal Structure and Pair Potentials: A Molecular-Dynamics Study. *Phys. Rev. Lett.* **45**, 1196-1199, doi:10.1103/PhysRevLett.45.1196 (1980).

13 Parrinello, M. & Rahman, A. Polymorphic transitions in single crystals: A new molecular dynamics method. *J. Appl. Phys.* **52**, 7182-7190, doi:10.1063/1.328693 (1981).
