# Supplementary material for: Monitoring the binding and insertion of a single transmembrane protein by an insertase
Source: Nat Commun. 2021 Dec 6;12:7082. doi: 10.1038/s41467-021-27315-3 (PMC8648943; doi:10.1038/s41467-021-27315-3)
Supplement: Supplementary file 3 — Description of Additional Supplementary Files [file 41467_2021_27315_MOESM3_ESM.pdf]

Description of Additional Supplementary Files

File Name: Supplementary Movie 1
Description: **Visualization of mechanical separation of Pf3 from YidC in the D18-R366 complex (Supplementary Fig. 7c, 9).** The movie shows the full unbinding process presented in snapshots in Supplementary Fig. 9. Pf3 (shown in red) is mechanically pulled by carbon Cα of F44 (highlighted as a sphere) along the membrane normal away from YidC (depicted in purple with CH2 colored grey). The position of the membrane is visualized by white spheres (phosphate groups).
Pulling velocity 0.2 m s^{-1}, simulation time 80 ns.
