# Supplementary material for: Monitoring the binding and insertion of a single transmembrane protein by an insertase
Source: Nat Commun. 2021 Dec 6;12:7082. doi: 10.1038/s41467-021-27315-3 (PMC8648943; doi:10.1038/s41467-021-27315-3)
Supplement: Supplementary file 5 — Reporting Summary [file 41467_2021_27315_MOESM5_ESM.pdf]

nature portfolio

Corresponding author(s): Daniel J. Müller

Last updated by author(s): Oct 26, 2021

Reporting Summary

Nature Portfolio wishes to improve the reproducibility of the work that we publish. This form provides structure for consistency and transparency in reporting. For further information on Nature Portfolio policies, see our Editorial Policies and the Editorial Policy Checklist.

Statistics

For all statistical analyses, confirm that the following items are present in the figure legend, table legend, main text, or Methods section.

n/a	Confirmed	
☐	☒	The exact sample size (n) for each experimental group/condition, given as a discrete number and unit of measurement
☐	☒	A statement on whether measurements were taken from distinct samples or whether the same sample was measured repeatedly
☐	☒	The statistical test(s) used AND whether they are one- or two-sided<br>*Only common tests should be described solely by name; describe more complex techniques in the Methods section.*
☐	☒	A description of all covariates tested
☒	☐	A description of any assumptions or corrections, such as tests of normality and adjustment for multiple comparisons
☐	☒	A full description of the statistical parameters including central tendency (e.g. means) or other basic estimates (e.g. regression coefficient) AND variation (e.g. standard deviation) or associated estimates of uncertainty (e.g. confidence intervals)
☐	☒	For null hypothesis testing, the test statistic (e.g. F, t, r) with confidence intervals, effect sizes, degrees of freedom and P value noted<br>*Give P values as exact values whenever suitable.*
☒	☐	For Bayesian analysis, information on the choice of priors and Markov chain Monte Carlo settings
☒	☐	For hierarchical and complex designs, identification of the appropriate level for tests and full reporting of outcomes
☒	☐	Estimates of effect sizes (e.g. Cohen's d, Pearson's r), indicating how they were calculated

Our web collection on statistics for biologists contains articles on many of the points above.

Software and code

Policy information about availability of computer code

Data collection	Bruker Nanoscope (versions 9.1 and 9.4R3), Burst-Analyzer (version 2.0), GROMACS (version 2018)
Data analysis	R (version 3.6.1), MATLAB (version 2018b), Origin (version 2019), PyMOL (version 2.5)

For manuscripts utilizing custom algorithms or software that are central to the research but not yet described in published literature, software must be made available to editors and reviewers. We strongly encourage code deposition in a community repository (e.g. GitHub). See the Nature Portfolio guidelines for submitting code & software for further information.

Data

Policy information about availability of data

All manuscripts must include a data availability statement. This statement should provide the following information, where applicable:

- Accession codes, unique identifiers, or web links for publicly available datasets
- A description of any restrictions on data availability
- For clinical datasets or third party data, please ensure that the statement adheres to our policy

The data that support the findings of this study are available from the corresponding author upon reasonable request.

Field-specific reporting

Life sciences study design

All studies must disclose on these points even when the disclosure is negative.

Sample size	The sample size was determined by calculating the number of adhesion events or simulations. The SMFS samples were taken from at least five independent experiments, with each experiment investigating a new biological sample preparation, buffer solution, AFM cantilevers, etc. Within each experiment several hundreds of force-distance curves were experimentally collected and analyzed.
Data exclusions	SMFS adhesion events weaker than five times the standard deviation of the baseline noise of the experiment (≈ 15 pN) were excluded from the analysis. Also, adhesion events between Pf3 and YidC, which occurred closer than 5 nm to the tip-sample contact point (to avoid taking into account non-specific tip-sample interaction events) and further than the pre-defined distance of 25 nm (to avoid taking into account macromolecular aggregates adhering to the cantilever tip) from the tip-sample contact point. Also force-distance curves containing multiple adhesion events were excluded as they likely originated from non-specific interactions. The pre-defined distance of 25 nm is determined based on the length of the PEG linker and the protein attached to the AFM tip. Furthermore, when unfolding reconstituted Pf3 from the membrane adhesion events detected in force-distance curves that were closer than 10 nm to the tip-sample contact point were excluded from the analysis to avoid the inclusion of non-specific interactions of the AFM tip with the sample. In addition, adhesion events detected in force-distance curves being further than 25 nm from the tip-sample contact point were excluded from analysis because the contour length of a fully stretched Pf3 is smaller than 25 mn.
Replication	Some attempts for SMFS experiments were unsuccessful due to failed membrane protein reconstitution or failed chemical modification of the AFM tip. To compensate for such unsuccessful experiments many experimental repetitions have been conducted. The adhesion forces analyzed orignate from at least 5 independent SMFS experiments at which the Pf3 had been succesfully chemically attached to the AFM tip.
Randomization	When multiple contact times were collected from an experimental sample, the acquisition of contact times was randomized. The SMFS experiments were repeated at least 5 independent times. Each of the YidC or other biological sample characterized had been clearly labelled and biochemically characterized before of the experiments.
Blinding	Blinding during data collection does not alter the experimental outcome because all microscopy settings were pre-set with changes only in the contact time or cantilever velocity as indicated. A control of the data analysis by a blinded researcher from outside of the list of authors confirmed the outcome of the analysis.

Reporting for specific materials, systems and methods

We require information from authors about some types of materials, experimental systems and methods used in many studies. Here, indicate whether each material, system or method listed is relevant to your study. If you are not sure if a list item applies to your research, read the appropriate section before selecting a response.

Materials & experimental systems

n/a	Involved in the study	
☒	☐	Antibodies
☒	☐	Eukaryotic cell lines
☒	☐	Palaeontology and archaeology
☒	☐	Animals and other organisms
☒	☐	Human research participants
☒	☐	Clinical data
☒	☐	Dual use research of concern

Methods

n/a	Involved in the study	
☒	☐	ChIP-seq
☒	☐	Flow cytometry
☒	☐	MRI-based neuroimaging
